# Perturbation-Based Balance Training to Improve Step Quality in the Chronic Phase After Stroke: A Proof-of-Concept Study

**DOI:** 10.3389/fneur.2018.00980

**Published:** 2018-11-22

**Authors:** Hanneke J. R. van Duijnhoven, Jolanda M. B. Roelofs, Jasper J. den Boer, Frits C. Lem, Rifka Hofman, Geert E. A. van Bon, Alexander C. H. Geurts, Vivian Weerdesteyn

**Affiliations:** ^1^Department of Rehabilitation, Donders Institute for Brain, Cognition and Behaviour, Radboud University Medical Center, Nijmegen, Netherlands; ^2^Department of Rehabilitation, Sint Maartenskliniek, Nijmegen, Netherlands; ^3^Rehabilitation Medical Centre Klimmendaal, Arnhem, Netherlands; ^4^Research, Sint Maartenskliniek, Nijmegen, Netherlands

**Keywords:** paresis, neurological rehabilitation, postural balance, exercise therapy, physical therapy modalities

## Abstract

**Introduction:** People with stroke often have impaired stepping responses following balance perturbations, which increases their risk of falling. Computer-controlled movable platforms are promising tools for delivering perturbation-based balance training under safe and standardized circumstances.

**Purpose:** This proof-of-concept study aimed to identify whether a 5-week perturbation-based balance training program on a movable platform improves reactive step quality in people with chronic stroke.

**Materials and Methods:** Twenty people with chronic stroke received a 5-week perturbation-based balance training (10 sessions, 45 min) on a movable platform. As the primary outcome, backward, and forward reactive step quality (i.e., leg angle at stepping-foot contact) was assessed with a lean-and-release (i.e., non-trained) task at pre-intervention, immediately post-intervention, and 6 weeks after intervention (follow-up). Additionally, reactive step quality was assessed on the movable platform in multiple directions, as well as, the percentage side steps upon sideward perturbations. To ensure that changes in the primary outcome could not solely be attributed to learning effects on the task due to repeated testing, 10 randomly selected participants received an additional pre-intervention assessment, 6 weeks prior to training. Clinical assesments included the 6-item Activity-specific Balance Confidence (6-ABC) scale, Berg Balance Scale (BBS), Trunk Impairment Scale (TIS), 10-Meter Walking Test (10-MWT), and Timed Up and Go-test (TUG).

**Results:** After lean-and-release, we observed 4.3° and 2.8° greater leg angles at post compared to pre-intervention in the backward and forward direction, respectively. Leg angles also significantly improved in all perturbation directions on the movable platform. In addition, participants took 39% more paretic and 46% more non-paretic side steps. These effects were retained at follow-up. Post-intervention, BBS and TIS scores had improved. At follow-up, TIS and 6-ABC scores had significantly improved compared to pre-intervention. No significant changes were observed between the two pre-intervention assessments (n=10).

**Conclusion:** A 5-week perturbation-based balance training on a movable platform appears to improve reactive step quality in people with chronic stroke. Importantly, improvements were retained after 6 weeks. Further controlled studies in larger patient samples are needed to verify these results and to establish whether this translates to fewer falls in daily life.

**Trial registration:** The Netherlands National Trial Register (NTR3804). http://www.trialregister.nl/trialreg/admin/rctview.aspTC=3804

## Introduction

Falls are among the most common complications after stroke (1). Post-stroke fall incidence rates vary between 1.4 and 5.0 falls each person-year (2). Falls are associated with worsening of functional outcomes post stroke (3). A vicious circle of falling, fear of falling, and inactivity can lead to further functional decline (2).

Impaired balance and gait capacities are the most important risk factors for falls after stroke (4, 5). Improving these capacities is, therefore, an important goal in rehabilitation. However, a Cochrane review on interventions for preventing falls after stroke did not show beneficial effects of exercise training aimed at improving balance and gait on fall rates (6). This is in contrast with the overwhelming evidence from the healthy elderly population, in which group- and home-based exercise programs do reduce fall rates and fall risk (7). The question arises whether the types of exercise training previously used in the stroke population are indeed suitable.

One important aspect that has yet received only limited attention in previous training programs for people with stroke is the role of reactive stepping responses while standing and walking (8–11). Following balance perturbations, fast and accurate stepping is an essential strategy to prevent falling (12, 13). People with stroke have an impaired capacity to execute such reactive stepping responses, particularly with the paretic leg (14–19). In fact, impaired stepping responses have been related to falling in people after stroke (20) and have shown to be predictive of fall risk after discharge from inpatient rehabilitation (21). Therefore, improving these reactive stepping responses following balance perturbations seems to be an important target for balance training after stroke.

Recent systematic literature reviews showed that perturbation-based balance training is effective to reduce fall risk in both healthy older adults and in people with Parkinson's disease (22, 23). In addition, a prospective cohort study showed lower fall rates for a group of participants in the subacute phase after stroke who received perturbation-based balance training during inpatient rehabilitation, when compared to a matched historical control group (11). Very recently, a first study on this type of training in the chronic phase after stroke has been published (24). In this study, the experimental group received therapist-induced balance perturbations and demonstrated improved reactive balance control when tested under the trained circumstances. Yet, no significant reduction in fall rate was observed compared to the control group. These observations call for further research on the generalizability of perturbation-based balance training to non-trained circumstances in people with chronic stroke. In addition, it may be that the effects of perturbation-based balance training can be enhanced by further increasing the intensity and unpredictability of the perturbations, thus providing a greater challenge for this group.

For delivering challenging perturbation-based balance training under safe and standardized circumstances, computer-controlled movable platforms [e.g., the Radboud Falls Simulator (RFS) (25)] are helpful. We here report the results of a proof-of-principle study to evaluate the effects of a 5-week training program on a movable platform, aimed at improving reactive step quality in multiple perturbation directions, and at enhancing side stepping upon sideward perturbations with the paretic and non-paretic leg. As a primary outcome, reactive step quality in the backward and forward directions was assessed with a lean-and-release (i.e., non-trained) task at pre-intervention, immediately post-intervention, and at 6 weeks follow-up. In addition, reactive step quality was assessed on the movable platform in multiple directions, as well as, the percentage side steps taken upon sideward perturbations. In the present study, we focused on community-dwelling people in the chronic phase after stroke, as in this phase no further neurological recovery should be expected (26). In addition, during the chronic phase, people are frequently exposed to balance perturbations in daily life. We hypothesized that our participants would show improved reactive step quality and enhanced side stepping after completion of a 5-week perturbation-based balance training program.

## Methods

### Participants

From the outpatient rehabilitation population of our university hospital, a total of 20 persons in the chronic phase (>6 months) after stroke were included. Participant characteristics are given in Table 1. They had to be able to stand and walk “independently” as defined by a Functional Ambulation Categories (FAC) score of 4 or 5 (27). Exclusion criteria were (1) other neurological or musculoskeletal conditions affecting balance; (2) health conditions in which physical exercise was contra-indicated; (3) use of psychotropic drugs or other medication negatively affecting balance; (4) severe cognitive problems [Mini Mental State Examination (MMSE) <24] (28); (5) persistent unilateral spatial neglect [Behavioral Inattention Test–Star Cancellation Test <44) (29)]; and (6) behavioral problems interfering with compliance to the study protocol. The study protocol was approved by the Medical Ethical Board of the region Arnhem-Nijmegen and all participants gave written informed consent in accordance with the Declaration of Helsinki. This study was registered in the Netherlands Trial Register (NTR number 3804, http://www.trialregister.nl/trialreg/admin/rctview.aspTC=3804).

**Table 1:** Characteristics of study participants (*n* = 20).

Sex (men/women, % men)	12/8, 60%
Age (years)^*^	60.1 (8.1)
Months since stroke^*^	50 (39.4)
Stroke type (ischemic/hemorrhagic, % ischemic)	12/8, 60%
Affected body side (left/right, % left)	12/8, 60%
Fall history (number of falls in previous year)^*^	1.6 (1.8)
MMSE (range: 0–30)^*^	27.8 (1.9)
QVT lateral malleolus affected side (range: 0–8)^*^	4.2 (2.2)
MI-LE (range: 0–100)^*^	63.3 (19.8)
FMA-LE (range: 0–100%)^*^	64.9 (17.7)
FAC (4/5, % FAC 4)	4/16, 20%

MMSE, mini mental state examination; QVT, quantitative vibration threshold; MI-LE, Motricity Index lower extremity; FMA-LE, Fugl-Meyer assessment lower extremity; FAC, Functional Ambulation Categories.

* *Values are presented in means (SD)*.

### Design and study protocol

**Figure 1:**
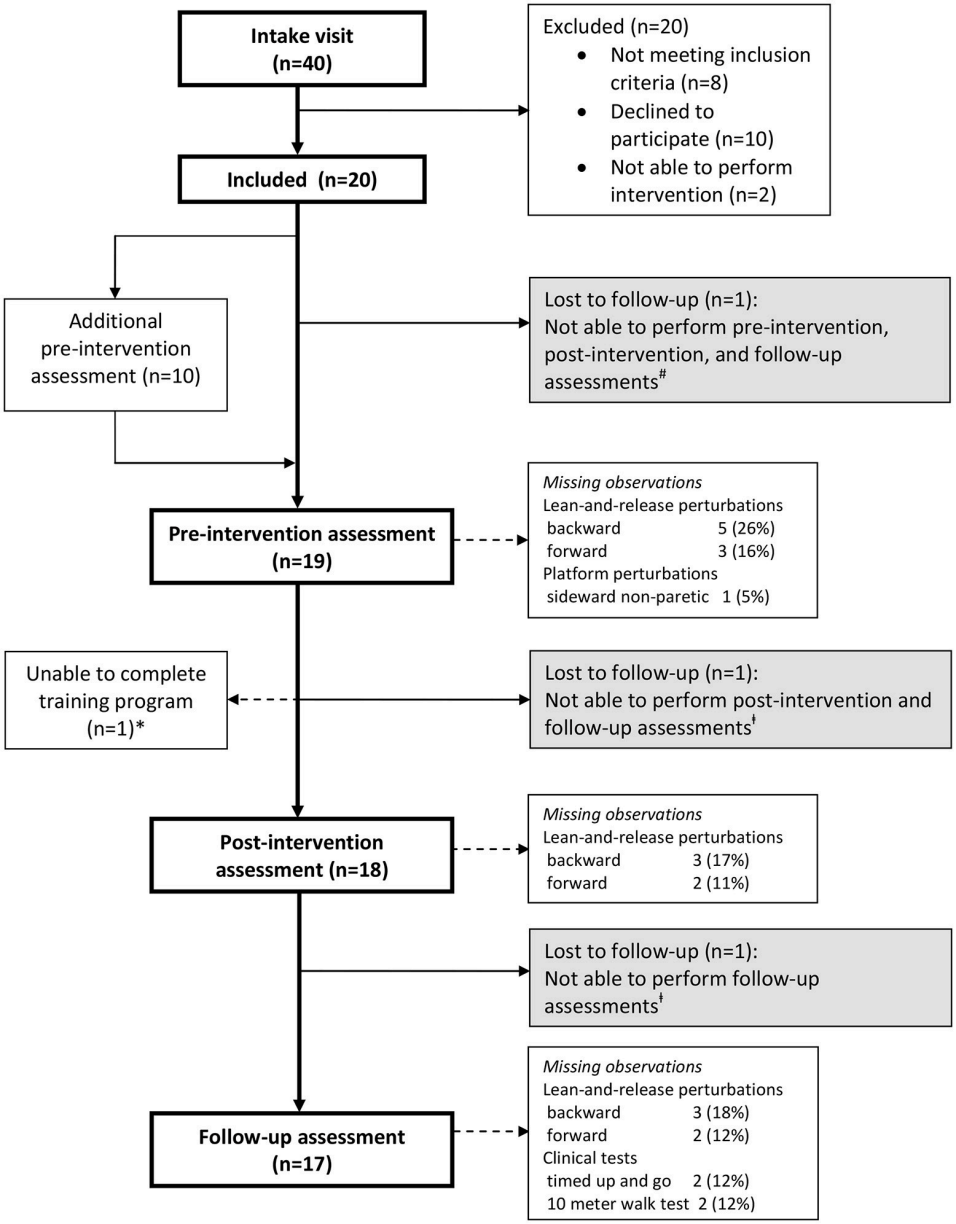
Flow of participants. ^*^One participant was able to complete only 3 out of 10 training sessions due to low back pain. Observations were included in all analyses according to the intention-to-treat principle. ^#^Lost to follow-up due to hip fracture after a fall, unrelated to the intervention. ^‡^Lost to follow-up due to illness, unrelated to the study.

We conducted a proof-of-principle study in which the participants received a 5-week perturbation-based balance training. Forty persons were invited for an intake visit to determine eligibility (Figure 1), and (after inclusion, *n* = 20) to determine participants' demographic and clinical characteristics [sex, age, months since stroke, type of stroke, affected body side, history of falls, quantitative vibration threshold (QVT) (30), Motricity Index lower extremity (MI-LE) (31), and Fugl-Meyer Assessment lower extremity (FMA-LE) (32)]. In addition, at the intake visit, initial training intensity was determined on the platform for each participant in each perturbation direction (see section Intervention). Thereafter, all assessments of reactive stepping, as well as, all clinical tests (see section Outcomes) were performed by each participant at pre-intervention, post-intervention (6 weeks after pre-intervention), and follow-up (12 weeks after pre-intervention). Yet, 10 participants (50%) who were randomly selected based on block randomization with stratification for severity of paresis (Motricity Index—Leg <64% vs. Motricity Index—Leg ≥64%), received an additional pre-intervention assessment of reactive stepping 6 weeks prior to the final pre-intervention assessment (see Figure 1). By comparing the results of both pre-intervention assessments in this subgroup, we were able to account for potential effects of repeated testing on reactive stepping. In the week after the (final) pre-intervention assessment, all participants started the 5-week perturbation-based balance training. More information about the study protocol as well as the raw data supporting the conclusions of this manuscript will be made available by the authors, without undue reservation, to any qualified researcher upon request.

### Intervention

The 5-week perturbation-based balance training program was delivered on the RFS [120 × 180 cm; Baat Medical, Enschede, The Netherlands (25)]. This movable platform can evoke reactive stepping responses by support-surface translations at magnitudes up to 4.5 m/s^2^ in any given horizontal direction. In designing our new training program we aimed to achieve a high intensity (i.e., number of perturbations), large variation (i.e., directions of perturbations), and high challenge (i.e., high perturbation magnitudes) of reactive stepping exercises, yet under safe circumstances. The use of this computerized technology allowed us to set these perturbation parameters in a highly standardized manner.

The selection of exercises in our program was inspired by the existing literature on reactive stepping responses in people with stroke and in healthy elderly (33–36). After a balance perturbation, people with stroke show a low step quality in all directions, with a tendency to use multiple steps (36), a slow execution of steps (36), and a preference to use the non-paretic leg (34, 35). Generally, the paretic leg shows difficulties both in executing a stepping response and in support limb control while stepping with the non-paretic side (17). For sideward perturbations, side stepping has proven to be a more efficient and effective strategy than using cross-over steps (37, 38), yet people with stroke and healthy elderly tend to prefer cross-over steps during recovery responses from sideward perturbations (18, 38). Therefore, the aim of the training program was to improve step quality after balance perturbations in eight different directions (forward, backward, both sideward, and four diagonal directions) by promoting the use of a single step, prevail side steps over cross-over steps, and enhance the speed of stepping. Reactive stepping was practiced both with the paretic and with the non-paretic leg. To achieve optimal results we used several previously reported and well known techniques like verbal feedback, blockage of the preferred leg, and stepping toward a target (39).

Participants received 45 min of training, two times a week, 5 weeks in a row, under supervision of a trained physiotherapist. During each session, participants received a total of 60–80 perturbations. During all exercises, participants were secured by a safety harness attached to a sliding rail on the ceiling. The level of difficulty was gradually increased across sessions based on a standardized protocol, yet based on an individualized initial training intensity in each perturbation direction, as determined during the intake visit. Initial training intensity was defined as the maximal intensity at which participants were able to restore their balance without taking a step, plus 0.25 m/s^2^. We progressed the difficulty of the training program by: (1) increasing the intensity of the perturbation; (2) increasing the unpredictability of the start and the direction of the perturbations across sessions; and (3) by adding dual tasks starting from training session 6 (see Tables 2, 3 for details on the content of the training program).

**Table 2:** Content of the perturbation-based balance training program.

**Session**	**Intensity of the perturbation**	**Predictability of the perturbation**	**Direction^#^**	**Additional training conditions**
1	Initial^*^	Direction indicated and countdown to perturbation onset	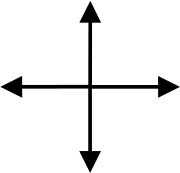
2	Initial	Direction indicated	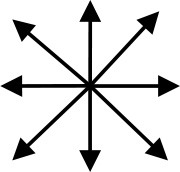
3	125% of initial	Direction indicated	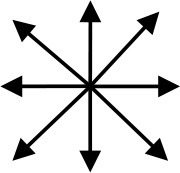
4	125% of initial	Random direction within blocks that contained perturbations in two directions [see graph: diagonal steps with paretic leg (blue) and non-paretic leg (red)]	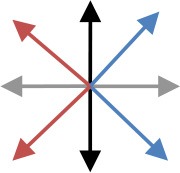
5	150% of initial	Random direction within blocks that contained perturbations in two directions [see graph: diagonal steps with paretic leg (blue) and non-paretic leg (red)]	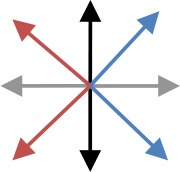
6	150% of initial	Random direction within blocks that contained perturbations in two directions [see graph: diagonal steps with either leg forward (purple) or backward (green)]	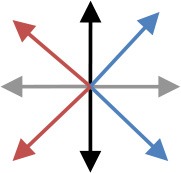	Cognitive dual task (visual Stroop task)
7	175% of initial	Random direction within blocks that contained perturbations in two directions (see graph for colored combinations)	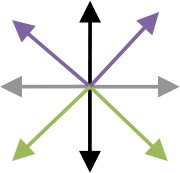	Motor dual task (marching in place)
8	175% of initial	All directions in random order	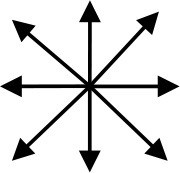	Cognitive dual task (visual Stroop task)
9	200% of initial	All directions in random order	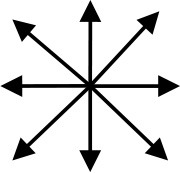	Motor dual task (marching in place)
10	200% of initial	All directions in random order	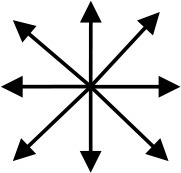	Combined cognitive and motor dual task

* The initial training intensity was the maximal intensity at which participants restored their balance without taking a step, plus 0.25 m/s^2^.

# *The arrows in the graph depict the perturbation direction (forward, backward, paretic, non-paretic, and four diagonal directions)*.

**Table 3:** Training intensities per session (m/s^2^) [Values are presented in means (SD)].

**Session**	**Forward perturbations**	**Backward perturbations**	**Perturbations toward paretic side**	**Perturbations toward non-paretic side**
1 and 2	1.02 (0.21)	0.83 (0.18)	1.06 (0.22)	1.10 (0.20)
3 and 4	1.28 (0.24)	1.05 (0.23)	1.35 (0.28)	1.41 (0.24)
5 and 6	1.53 (0.30)	1.28 (0.26)	1.61 (0.31)	1.67 (0.29)
7 and 8	1.80 (0.34)	1.49 (0.31)	1.88 (0.39)	1.94 (0.35)
9 and 10	2.04 (0.41)	1.67 (0.36)	2.14 (0.44)	2.21 (0.40)
Multiple stepping threshold	3.03 (1.36)	2.21 (0.88)	1.78 (0.90)	1.68 (0.96)

The 10 participants who performed two pre-intervention assessments were allowed to continue usual care during the 6 weeks in between these assessments, including any kind of physical therapy (if applicable). All participants were asked to refrain from additional balance exercises at home during the training period, but were free to receive (or continue) usual care during the follow-up.

### Reactive stepping assessment

During pre, post, and follow-up assessments, reactive stepping was recorded following two types of balance perturbations: lean-and-release perturbations (backward and forward directions) and platform perturbations (backward, forward, sideward paretic, sideward non-paretic directions). During all recordings, participants wore their own shoes and stood at a fixed foot position with a distance of 4.5 cm between the medial sides of both feet. They wore a safety harness (attached to a sliding rail on the ceiling) to prevent them from falling, but which did not provide body (weight) support.

The lean-and-release task is a frequently used experimental paradigm for studying reactive stepping responses (21, 35, 40). Importantly, this type of perturbation was not trained and was, therefore, selected as the primary outcome. Using different types of perturbation for training and assessment is in line with a previous study on perturbation-based balance training (33). Participants were instructed to lean into the tether at an inclincation angle of 10° and, upon its unexpected release, to recover their balance by taking a single step. They were free to select which leg they used for stepping. After several practice trials, 10 outcome trials were recorded, five trials in the backward and five trials in the forward direction.

During the platform perturbation trials, participants received unpredictable and sudden horizontal translations in the forward, backward, sideward paretic, and sideward non-paretic directions. They were instructed to recover their balance with a single step. Perturbations consisted of an acceleration (300 ms), constant velocity (500 ms), and deceleration phase (300 ms). During the first assessment, the perturbation intensity was gradually increased with increments of 0.25 m/s^2^ until the participants reached the maximum intensity at which they were able to recover their balance with one step (multiple stepping threshold, maximal 4.5 m/s^2^). During the subsequent assessments, we used a fixed protocol with random perturbations in each direction, until the individual multiple stepping threshold (as determined during the first assessment) was reached. During all assessments, we ultimately recorded six outcome trials, three trials at the multiple stepping threshold and another three trials at one level (+0.125 m/s^2^) above this intensity.

In addition to these formal assessments, we also monitored progress of participants' reactive step quality on the platform across training sessions (sessions 1, 4, 7, and 10). Due to time limitations or technical problems, we managed to do this in full for 14 of the 20 participants. These participants received additional platform perturbations after the training session, alternating in the forward and backward directions, at the maximum of their capacity (i.e., 0.125 m/s^2^ above their individual multiple stepping threshold).

Reactive stepping responses were recorded at 100 Hz using an 8-camera 3D motion capture system (Vicon Motion Systems, Oxford, UK). Reflective markers were placed on anatomical landmarks according to the Vicon Plug-in-Gait model (41). An additional reflective marker was placed on the translating platform to correct marker positions for platform movement. Marker trajectory data were filtered with a second order, 5 Hz low-pass, zero-lag Butterworth filter.

**Figure 2:**
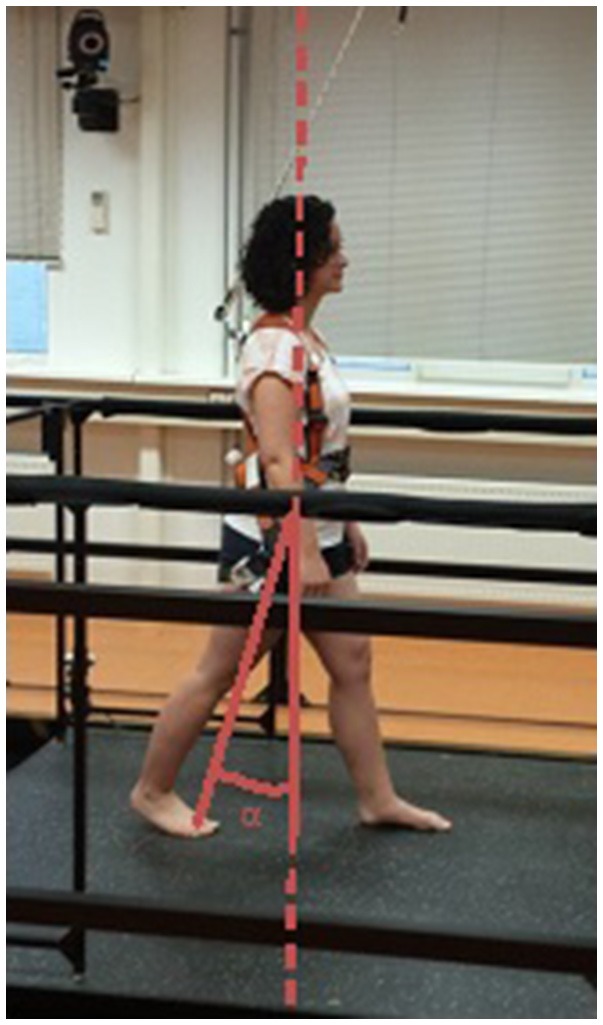
Definition of the leg angle. Reactive step quality was expressed as the leg angle (α) at stepping-foot contact. This figure shows the leg angle for a backward step.

From these data, we assessed the quality of the reactive step. This step quality is typically determined by how far the stepping foot is placed away from the centre of mass (CoM) into the direction of the induced loss of balance (15, 42–45). We expressed this foot-to-CoM relationship as the leg angle at first stepping-foot contact (Figure 2). In previous studies, the leg angle at stepping foot contact accurately distinguished between falls and successful recovery in healthy young subjects (43) and between single and multiple reactive steps in older women (42) and stroke survivors (19). The leg angle was defined as the angle between the vertical and the line connecting the mid-pelvis to the second metatarsal (backward and forward perturbations) or to the lateral malleolus (sideward perturbations) of the stepping foot. Larger positive leg angles correspond to better step quality.

### Outcomes

The primary outcome was reactive step quality (i.e., leg angle at first stepping-foot contact) following lean-and-release perturbations in the backward and forward directions. The reactive step quality in four directions and the proportion of side steps upon sideward perturbations on the platform were used as secondary outcome measures. In addition, several clinical tests were performed, namely: (1) the 6-item short version of the Activity-specific Balance Confidence scale (6-ABC; range: 0–100%) to assess the balance confidence for performing daily-life activities (46); (2) the Berg Balance Scale (BBS; range: 0–56) to test balance performance during activities of varying difficulty (47); (3) the Trunk Impairment Scale (TIS; range: 0–23) to evaluate static and dynamic sitting balance and coordination of trunk movement (48); (4) the 10-Meter Walking Test at comfortable walking speed (10-MWT); and (5) the Timed Up and Go test (TUG) to quantify functional mobility (49). To determine the ability of participants to recover balance according to the instructions (i.e., with a single step), we also calculated the success rate for the lean-and-release and platform perturbations.

### Statistical analysis

We first verified by means of Generalized Estimated Equations modeling (GEE, autoregressive correlation structure) that potentially confounding variables [i.e., initial inclination angles for lean-and-release perturbations, the stepping leg (paretic/non-paretic), the maximal percentage of body weight supported by the harness system, and the angle of the trunk with the vertical at first stepping-foot contact] were not different between pre-intervention, post-intervention, and follow-up for the primary outcome assessments. As these analyses yielded no significant effects of time, we did not include these variables in further analyses.

To study changes in the primary and secondary outcomes (i.e., leg angles, percentages side steps, clinical scales) following training, we conducted a GEE analysis with time (pre-intervention, post-intervention, and follow-up) as within-subject factor. Furthermore, for the subgroup of participants who received two pre-intervention assessments, we determined the effects of repeated testing on reactive stepping by means of paired *t*-tests. Statistical analyses were performed with SPSS (version 22.0). *P* < 0.05 was considered statistically significant.

## Results

The inclusion flow diagram (Figure 1) shows the numbers (and reasons) of participants who were lost to follow-up, as well as, any missing observations at the assessments. No intervention-related adverse events were reported. Table 4 shows a detailed overview of all outcome measures at pre-intervention, post-intervention, and follow-up (*n* = 20), together with the reactive stepping data for the two pre-intervention assessments (*n* = 10). Participants were not always able to regain balance with a single step. Table 5 shows the percentage of successful lean-and-release and platform perturbation trials at pre-intervention, post-intervention, and follow-up.

**Table 4:** Primary and secondary outcome measures.

	**Pre-intervention** **Mean (SE)** **(*n* = 20)**	**Post-intervention** **Mean (SE)** **(*n* = 20)**	**Follow-up** **Mean (SE)** **(*n* = 20)**	**Main effect of time** **(*p*-value)** **(*n* = 20)**	**First pre-intervention** **Mean (SE)** **(*n* = 10)**	**Final pre-intervention** **Mean (SE)** **(*n* = 10)**	**Main effect of time** **(*p*-value)** **(*n* = 10)**
**PRIMARY OUTCOMES**							
**Lean-and-release perturbations**							
Backward leg angle (°)	0.3 (1.2)	4.6 (1.3)	4.0 (1.2)	0.001	−1.6 (1.7)	−0.8 (1.2)	0.589
Forward leg angle (°)	22.4 (0.8)	25.2 (0.5)	25.1 (0.7)	<0.001	23.0 (1.1)	23.0 (1.2)	0.987
**SECONDARY OUTCOMES**							
**Platform Perturbations**							
Backward leg angle (°)	−2.0 (1.4)	2.1 (1.1)	2.5 (0.8)	0.001	−3.5 (1.7)	−3.2 (1.5)	0.730
Forward leg angle (°)	21.1 (0.8)	23.6 (0.5)	23.3 (0.7)	0.001	20.1 (1.5)	20.4 (0.8)	0.699
Paretic side step (%)	19 (8)	59 (11)	58 (11)	0.001	29 (15)	30 (15)	0.907
Non-paretic side step (%)	37 (10)	84 (6)	80 (7)	<0.001	21 (14)	38 (16)	0.374
Paretic side step leg angle (°)^*^	17.6 (1.5)	19.8 (1.3)	19.4 (1.2)	0.012		
Non-paretic side step leg angle (°)^*^	17.6 (0.7)	18.8 (0.6)	19.7 (0.5)	0.001		
**Clinical Scales**							
BBS	52.4 (0.9)	53.3 (0.7)	52.7 (0.8)	0.047		
TIS	16.1 (0.6)	17.9 (0.7)	16.7 (0.6)	<0.001		
6-ABC	41.5 (5.7)	45.1 (4.8)	49.4 (5.6)	0.014		
Comfortable walking speed (km/h)	3.5 (0.2)	3.7 (0.2)	3.6 (0.2)	0.127		
TUG (s)	10.4 (0.8)	10.8 (0.8)	10.0 (0.8)	0.307		

Estimated marginal means (standard errors) for leg angles, percentage side steps, and clinical scales at pre-intervention, post-intervention, and follow-up for the total study sample (n = 20) and mean values (standard errors) for leg angles and percentage side steps at the first and final pre-intervention assessment (n = 10).

* *Only three participants who received two pre-intervention assessments (n = 10) took a paretic side step at both assessments, whereas none of the participants took a non-paretic side step at both assessments. Therefore, sideward leg angles were not compared between pre-intervention assessments. BBS, Berg Balance Scale (range: 0–56); TIS, Trunk Impairment Scale (range: 0–23); 6-ABC, 6-item short version of the Activity-specific Balance Confidence scale (range: 0–100%); TUG, Timed Up and Go test*.

**Table 5:** Percentage (SD) of trials recovered with a single step.

	**Pre-intervention**	**Post-intervention**	**Follow-up**
**LEAN-AND-RELEASE PERTURBATIONS**			
Backward	64 (44)	73 (42)	80 (39)
Forward	73 (41)	82 (33)	81 (35)
**PLATFORM PERTURBATIONS**			
Backward	38 (24)	81 (23)	85 (25)
Forward	56 (35)	92 (18)	93 (14)
Toward paretic side^*^	28 (21)	66 (41)	69 (37)
Toward non-paretic side^*^	36 (32)	92 (12)	80 (25)

* *N.B. This concerns both side steps and cross-over steps*.

### Lean-and-release perturbations

**Figure 3:**
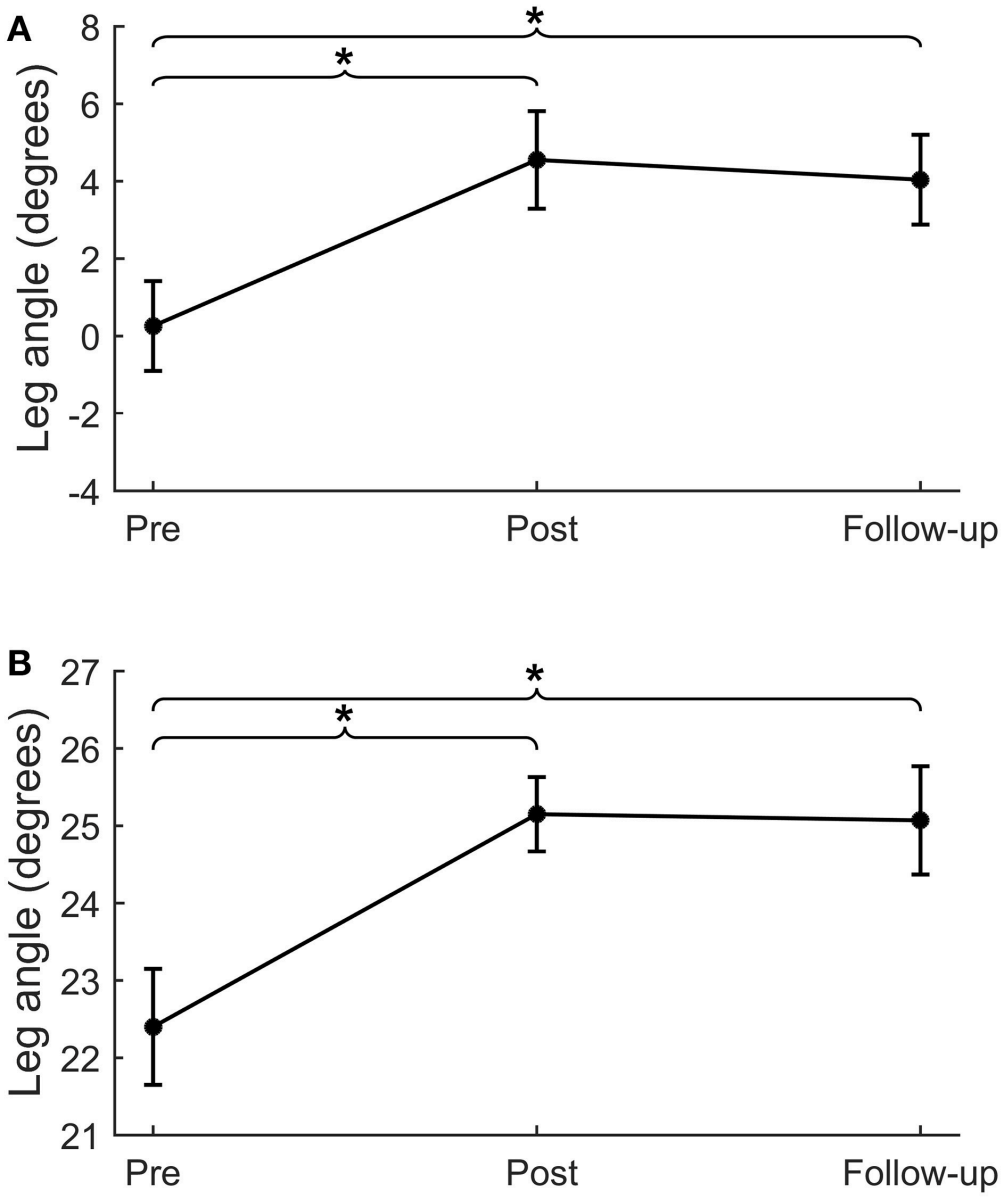
Lean-and-release perturbations. Step quality (i.e., leg angle) for lean-and-release perturbations in the backward **(A)** and forward **(B)** directions. *p < 0.01.

Backward and forward leg angles at post-intervention were significantly larger compared to pre-intervention (backward: Δ_pre−post_ = 4.3 ± 1.3°, *p* = 0.001; forward: Δ_pre−post_ = 2.8 ± 0.7°, *p* < 0.001; Figure 3 and Table 4). These larger leg angles were retained six weeks after training at follow-up (backward: Δ_pre−fu_ = 3.8 ± 1.2°, *p* = 0.001; forward: Δ_pre−fu_ = 2.7 ± 0.6°, *p* < 0.001). For both directions, leg angles were not different between the two pre-intervention assessments.

### Platform perturbations

**Figure 4:**
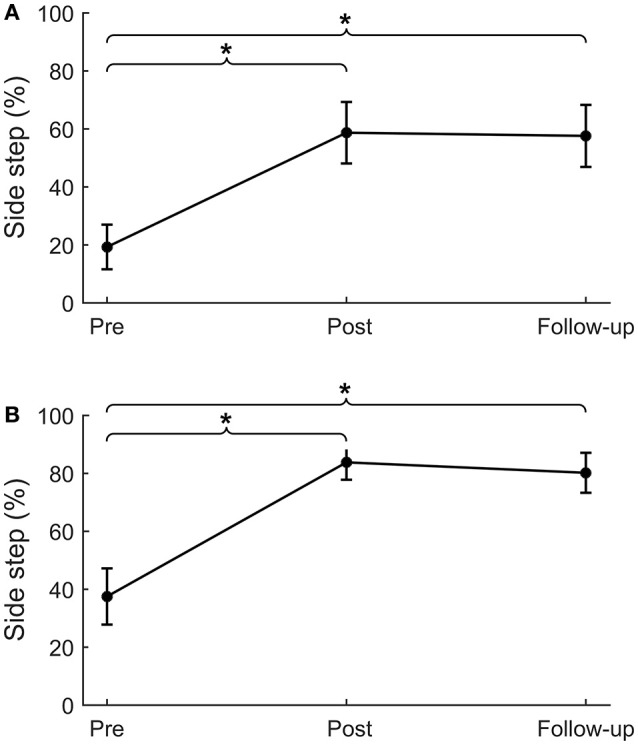
Sideward platform perturbations. Percentage of side steps for platform perturbations in the sideward paretic **(A)** and sideward non-paretic **(B)** directions. *p < 0.01.

For backward and forward platform perturbations, leg angles at post-intervention were significantly larger compared to pre-intervention (backward: Δ_pre−post_ = 4.1 ± 1.2°, *p* = 0.001; forward: Δ_pre−post_ = 2.5 ± 0.8°, *p* = 0.001; Table 4). This difference in leg angle was retained at follow-up (backward: Δ_pre−fu_ = 4.5 ± 1.2°, *p* < 0.001; forward: Δ_pre−fu_ = 2.2 ± 0.6°, *p* < 0.001). For sideward perturbations, the percentage of paretic and non-paretic side steps increased from pre- to post-intervention (paretic: Δ_pre−post_ = 39 ± 12%, *p* = 0.001, Figure 4A; non-paretic: Δ_pre−post_ = 46 ± 9%, *p* < 0.001, Figure 4B). This effect was also retained at follow-up (paretic: Δ_pre−fu_ = 38 ± 11%, *p* = 0.001; non-paretic: Δ_pre−fu_ = 43 ± 9%, *p* < 0.001). Furthermore, the number of participants who took at least one side step had increased after training. Before the start of the intervention, six and ten participants took one or more side steps with the paretic and non-paretic leg, respectively, which numbers increased to 12 and 17 at the post-intervention assessment. For side steps toward the paretic side, leg angles increased from pre- to post-intervention (Δ_pre−post_ = 2.3 ± 0.8°, *p* = 0.004), which effect was retained at follow-up (Δ_pre−fu_ = 1.9 ± 0.7°, *p* = 0.006). For side steps toward the non-paretic side, leg angles at post-intervention were not significantly larger compared to pre-intervention, although a trend toward larger leg angles was visible (Δ_pre−post_ = 1.2 ± 0.7°, *p* = 0.075). Six weeks after training, at follow-up, non-paretic leg angles were significantly larger compared to pre-intervention (Δ_pre−fu_ = 2.1 ± 0.6°, *p* = 0.001).

### Clinical tests

Participants had a slightly higher BBS score at post compared to pre-intervention (Δ_pre−post_ = 0.9 ± 0.4, *p* = 0.021), however, this effect was not retained at follow-up (Δ_pre−fu_ = 0.4 ± 0.6, *p* = 0.493). Yet, TIS scores at post-intervention and at follow-up were significantly higher compared to pre-intervention (Δ_pre−post_ = 1.8 ± 0.5, *p* < 0.001; Δ_pre−fu_ = 0.7 ± 0.3, *p* = 0.022). The difference in 6-ABC scores between pre- and post-intervention did not reach statistical significance (Δ_pre−post_ = 3.7 ± 3.1, *p* = 0.240), however, at follow-up, participants rated their balance confidence significantly higher compared to pre-intervention (Δ_pre−fu_ = 7.9 ± 2.9, *p* = 0.007). For comfortable walking speed and TUG no significant differences were found between pre-intervention, post-intervention, or follow-up.

### Progress of step quality

**Figure 5:**
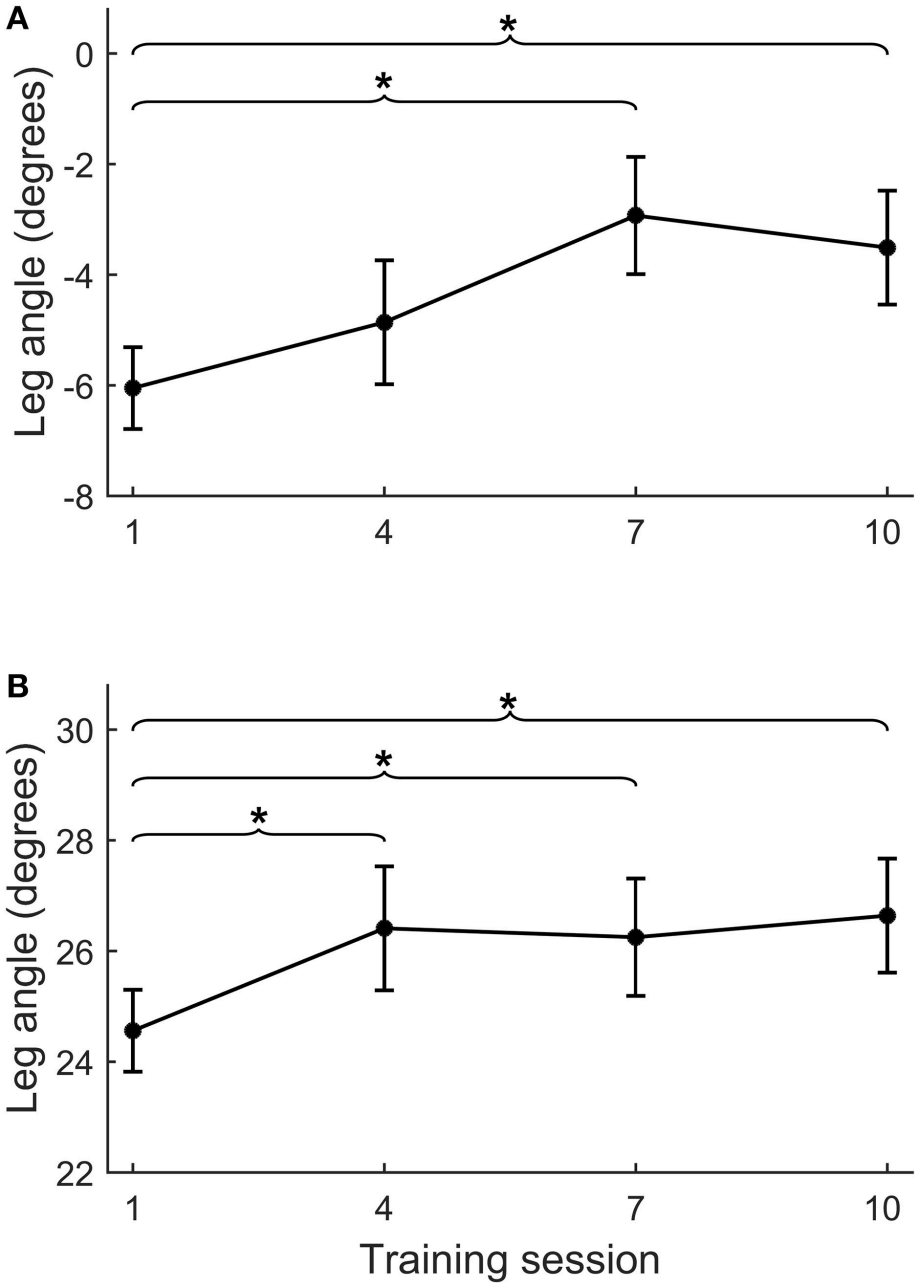
Course of leg angle improvement across training sessions. Course of improvement in step quality (i.e., leg angle) across training sessions 1, 4, 7, and 10 for backward **(A)** and forward **(B)** platform perturbations. For practical reasons, we placed the reflective markers during the training sessions on the feet and L4 vertebra, instead of on the feet and both spina iliaca as was done during the formal assessments. Therefore, leg angles from the training sessions and assessments are not directly comparable. *p < 0.05.

During the intervention period, backward leg angles increased from training session 1–7 (Δ_s1−s7_ = 3.4 ± 4.8°, *p* = 0.041), but did not further increase at subsequent sessions. The forward leg angle increased from session 1–4 (Δ_s1−s4_ = 1.8 ± 2.6°, *p* = 0.033), but did not further increase at subsequent sessions (Figure 5).

## Discussion

This proof-of-principle study investigated whether a 5-week perturbation-based multidirectional balance training program was able to improve reactive stepping in 20 persons in the chronic phase after stroke. In accordance with our hypotheses, we found that after completion of the training program reactive step quality had improved in the backward, forward, and both sideward directions. In addition, both paretic and non-paretic side steps were more frequently used for recovering balance upon sideward perturbations. Both types of effect were retained at follow-up. Several clinical scales showed significant immediate (BBS, TIS) and/or delayed (TIS, 6-ABC) training effects as well, albeit relatively small sized compared to the assessments of reactive stepping.

The hypothesis that our perturbation-based balance training would improve step quality in people with chronic stroke was corroborated by an increase in leg angle at first stepping-foot contact following lean-and-release perturbations of 4.3 (backward) and 2.8 degrees (forward) between pre- and post-intervention. This parameter has previously been shown to be a valid indicator of reactive step quality, as it accurately distinguished between successful (no fall) and unsuccessful (fall) recovery following large balance perturbations (43) and between single and multiple stepping in elderly individuals and people with stroke (19, 42). At post-intervention, the leg angles during the platform perturbations showed similar improvements as those observed during lean-and-release perturbations (4.1 and 2.5 degrees in the backward and forward directions, respectively). As the lean-and-release perturbations were not included in the training program, we conclude that generalization of the training effects to non-trained tasks has occurred, which implies “real” training effects. Importantly, these improvements were retained for both types of perturbations after a period of 6 weeks during which no further practice of reactive stepping took place. Although these results are promising, it remains unknown for how long the observed improvements in reactive stepping are retained after this follow-up period of 6 weeks.

The notion that perturbation-based balance training can improve reactive stepping is in accordance with a previous study in community-dwelling older adults (33). After 6 weeks (18 sessions of 30 min) of perturbation training, these elderly persons more frequently used single stepping responses and had less foot collisions during sideward perturbations. Since foot collisions mostly occur during cross-over steps, the reduction in collisions is in line with our observation of more side steps being taken after the training. Only one case study in a sub-acute stroke patient (9) has previously been published on training-induced improvements in reactive step quality. This study demonstrated increased effectiveness of reactive stepping responses and increased use of the paretic leg for stepping after targeted perturbation training. As the training was provided in the sub-acute phase, it remained unknown whether improved reactive stepping was caused by spontaneous neurological recovery, by training-induced functional recovery, or by a combination of both. Although, generally, neurological (motor) recovery cannot be expected beyond 3 months after stroke (50), functional recovery can still be reached in the chronic phase (51). In this study, we indeed found that targeted balance training resulted in (further) functional recovery in a relatively high functioning group of chronic stroke patients. During training, participants were “forced” to step with both the paretic and non-paretic leg in response to challenging perturbations. This type of training may unmask latent motor capacity, especially of the proximal leg and trunk muscles, that may have decayed as a result of stroke. This process may be based on the same mechanisms that underlie the effects of constrained induced movement therapy of the upper limb, but the exact neurophysiological mechanisms are a relevant topic for further research. As there are no established methods for measuring reactive step quality in people with stroke, the clinical relevance of the presently observed improvements remains to be identified. Nevertheless, in young adults an increase in leg angle of just 1 degree resulted in a three-fold greater odds of successfully recovering from a large backward perturbation (43). Hence, we conclude that the presently observed improvements in step quality are substantial, and most likely clinically relevant.

Although our training period of 5 weeks appears sufficiently long to achieve gains in reactive stepping, we raise the question whether there might have been further room for improvement. In the first training session, we conservatively chose a perturbation intensity only slightly above the individual stepping thresholds. Perturbation intensity was gradually increased each week according to a pre-defined protocol. Yet, it turned out that in the anteroposterior directions, the average perturbation intensities in the final training sessions were still below the participants' multiple stepping thresholds, as measured during the first balance assessment (see Table 3). In addition, we monitored progress in forward and backward step quality (across sessions 1, 4, 7, and 10) and observed a plateau from session 4 onwards for forward and from session 7 onwards for backward step quality (see Figure 5). These observations indicate that we have been rather conservative in progressing the level of difficulty across training sessions. Hence, for future application of the training protocol, we suggest to further challenge the participants by including greater increments in perturbation intensities.

The improvements that we observed in reactive stepping were accompanied by a perceived increase in balance confidence (as shown by the higher scores on the 6-ABC at follow-up). Yet, we found only minor and transient gains on the BBS as a clinical balance test, which were not considered clinically relevant. In the remaining clinical outcomes, we found no significant changes, except for a modest improvement in TIS scores at post-intervention and follow-up. These observations are in line with a recent study on perturbation-based balance training in people in the sub-acute phase after stroke, which resulted in a reduction in fall risk and fall rates compared to a historical cohort, yet without between-group differences on clinical test outcomes (11). One reason for this apparent discrepancy may be that the clinical tests included in our study do not capture (improvements in) reactive stepping, being the primary aim of our perturbation-based training program. Indeed, Innes et al. (52) found a wide range in BBS scores across stroke participants, which did not correspond to their level of reactive stepping capacity. Another explanation may be that our participants already had near-maximum BBS scores (median: 54, range: 42–56) at pre-intervention, leaving little room for further improvement. Yet, they did have impairments in reactive stepping, as their multiple stepping thresholds (i.e., the maximum perturbation intensity that could be sustained with a single step) were substantially lower than in healthy peers. For example, backward multiple stepping thresholds in our participants were 2.2 vs. 3.5 m/s^2^ in healthy peers (18). Hence, it seems that for our group of community ambulators after stroke (comfortable walking speed >0.8 m/s in 16 of the 19 participants), the ceiling effect in BBS scores results in an underestimation of their balance impairments. Therefore, in future studies on perturbation-based training, we recommend to consider alternative clinical balance tests that do include an assessment of reactive balance control. The mini-BEST, for instance, includes reactive stepping tests and also has a smaller ceiling effect than the BBS (53), which may further add to its suitability for community ambulators.

In this study, the use of the RFS had the advantage of delivering a training program that was standardized, safe, challenging and of high intensity. Yet, it should be mentioned that this type of technology is not yet widely available and future developments should be targeted at designing cheaper and more easy-to-use training devices for perturbation-based balance training. Another limitation of our study is that the leg angle at first stepping-foot contact did not provide insight into which part of the stepping response specifically responded to the perturbation-based balance training. Such insight could further enhance our understanding of functional balance recovery after stroke and, thereby, help optimize rehabilitation strategies for this patient group. Although the fact that we did not include a control group is an obvious limitation, a subgroup of 10 participants showed no differences in reactive stepping between the first and final pre-intervention assessments (before the start of the training). This result supports the notion that the observed improvements in reactive stepping after training are attributable to the perturbation-based balance training. Another limitation is the predictability of perturbation direction during the lean-and-release task. In the backward and forward platform perturbations, however, we found similar improvements in leg angles after training compared to the lean-and-release task. As perturbation direction during the platform perturbations was randomized across four different directions (backward, forward, sideward paretic, and sideward non-paretic), it appears that improvements in reactive stepping are not solely attributable to anticipation of participants.

Although we found improvements in reactive step quality after perturbation-based balance training, we did not evaluate whether our training program also contributed to fewer falls in daily life. Previous research showed, however, that impaired quality of reactive steps is related to increased fall rates during inpatient stroke rehabilitation (20), and that perturbation-based training in the sub-acute phase can reduce fall risk (11). These findings suggest that perturbation-based balance training may be an effective intervention for reducing fall rates, not only in the sub-acute phase but also in the chronic phase after stroke (10). Our chronic stroke participants were able to apply the learned stepping responses in non-trained circumstances and, importantly, retained their improvements in reactive stepping over a 6-week period without further practice. Therefore, perturbation-based balance training appears promising for improving the ability to recover from balance perturbations outside the laboratory or clinical setting (for example while experiencing trips or slips in daily life). Yet, further controlled studies in larger patient samples are needed to verify our results and to establish whether an improved step quality indeed translates to fewer falls in daily life.

## Author contributions

HvD, AG, and VW: study conception and design. HvD, JdB, FL, and AG: eligibility assessment. HvD, JR, GvB, and RH: data collection. HvD, JR, AG, and VW: analysis and interpretation. AG and VW: funding and supervision. HvD, JR, and VW: drafting of the manuscript. HvD, JR, JdB, FL, GvB, RH, AG, and VW: critical revision.

### Conflict of interest statement

The authors declare that the research was conducted in the absence of any commercial or financial relationships that could be construed as a potential conflict of interest. The reviewer AP and handling Editor declared their shared affiliation at the time of the review.
